# Dental Depot at Louisville

**Published:** 1852-01

**Authors:** 


Dental Depot at Louisville, Ky.—We see from a circular of Messrs. Sutcliffe, McAllister & Co., druggists, 524 Main street, Louisville, that they have supplied themselves with an extensive assortment of dental instruments, materials, teeth, &c., &c. We see on their list Jones, White & Co.'s teeth, Philadelphia, and J. Denhard's, Cincinnati. Also, gold and tin foil, gold plate, springs, wire, forceps, plugging instruments, excavators, files, emery wheels, brushes, and indeed almost every thing needed by the profession. We have no doubt these gentlemen are rendering the profession an essential service, and we hope they may receive that support which their integrity and attention to business so justly merits. They are agents for the Register; also, for the Journal, Recorder, and News Letter.

